# Zebrafish null mutants of Sept6 and Sept15 are viable but more susceptible to *Shigella* infection

**DOI:** 10.1002/cm.21750

**Published:** 2023-03-14

**Authors:** Vincenzo Torraca, Magdalena K. Bielecka, Margarida C. Gomes, Dominik Brokatzky, Elisabeth M. Busch‐Nentwich, Serge Mostowy

**Affiliations:** ^1^ Department of Infection Biology London School of Hygiene & Tropical Medicine London UK; ^2^ School of Life Sciences University of Westminster London UK; ^3^ Department of Medicine, Cambridge Institute of Therapeutic Immunology & Infectious Disease (CITIID) University of Cambridge Cambridge UK; ^4^ School of Biological and Behavioural Sciences, Faculty of Science and Engineering Queen Mary University of London London UK

**Keywords:** cytoskeleton, genetic compensation, null mutant, septins, *Shigella*, zebrafish

## Abstract

Septins are evolutionarily conserved GTP‐binding proteins known for their roles in cell division and host defence against *Shigella* infection. Although septin group members are viewed to function as hetero‐oligomeric complexes, the role of individual septins within these complexes or in isolation is poorly understood. Decades of work using mouse models has shown that some septins (including SEPT7) are essential for animal development, while others (including SEPT6) are dispensable, suggesting that some septins may compensate for the absence of others. The zebrafish genome encodes 19 septin genes, representing the full complement of septin groups described in mice and humans. In this report, we characterise null mutants for zebrafish Sept6 (a member of the SEPT6 group) and Sept15 (a member of the SEPT7 group) and test their role in zebrafish development and host defence against *Shigella* infection. We show that null mutants for Sept6 and Sept15 are both viable, and that expression of other zebrafish septins are not significantly affected by their mutation. Consistent with previous reports using knockdown of Sept2, Sept7b, and Sept15, we show that Sept6 and Sept15 are required for host defence against *Shigella* infection. These results highlight *Shigella* infection of zebrafish as a powerful system to study the role of individual septins in vivo.

## 1 INTRODUCTION

Septins are an enigmatic component of the cytoskeleton discovered from their role in yeast cell division (Mostowy & Cossart, 2012; Spiliotis & Nakos, 2021; Woods & Gladfelter, 2021). In humans, septins are organised into four groups based on amino acid sequence similarity: the SEPT2 group (SEPT1, SEPT2, SEPT4, SEPT5), SEPT3 group (SEPT3, SEPT9, SEPT12), SEPT6 group (SEPT6, SEPT8, SEPT10, SEPT11, SEPT14), and SEPT7 group (SEPT7). Members of each septin group assemble into hetero‐oligomeric complexes (including SEPT2–SEPT6–SEPT7–SEPT9–SEPT9–SEPT7–SEPT6–SEPT2) and higher‐order structures (including filaments, rings and cage‐like structures). In the case of bacterial infection, septins are recognised for their role in host defence against the human bacterial pathogen *Shigella flexneri* (Robertin & Mostowy, 2020; Torraca & Mostowy, 2016; van Ngo & Mostowy, 2019).

Animal models available to study septin biology in vivo are lacking. Decades of work using mouse models have shown that some septins (including SEPT7) are essential for animal development, while others (including SEPT6) are dispensable, suggesting that some septins may compensate for the absence of others (Ageta‐Ishihara et al., 2013; Kinoshita, 2008; Lassen et al., 2013; Menon et al., 2014; Mostowy & Cossart, 2012). Zebrafish are a valuable vertebrate model, historically used to study developmental processes because larvae are optically accessible and develop rapidly. Since zebrafish larvae emerged as a powerful model to study the development of innate immunity (Herbomel, Thisse, & Thisse, 1999), they have often been used to study the role of evolutionarily conserved protein families in the immune response. For example, the role of chemokines and chemokine receptors in response to infection has been explored in zebrafish using both transient knockdown and null mutant models (Cambier, O'Leary, O'Sullivan, Keane, & Ramakrishnan, 2017; Sommer, Torraca, Kamel, Lombardi, & Meijer, 2020; Sommer, Torraca, & Meijer, 2020; Torraca et al., 2015; Torraca, Otto, Tavakoli‐Tameh, & Meijer, 2017). In the case of septins, the zebrafish genome encodes 19 septin genes that are highly homologous to mouse and human septins, where all septin groups (SEPT2, SEPT3, SEPT6, and SEPT7 groups) are represented (Torraca & Mostowy, 2016; van Ngo et al., 2023). Because the immune system of zebrafish shares extensive genomic homology with the immune system of humans, zebrafish are a valuable model to study infection to a wide variety of human pathogens (Gomes & Mostowy, 2020; Torraca & Mostowy, 2018), including the cellular microbiology paradigm *S*. *flexneri* (Duggan & Mostowy, 2018; Schnupf & Sansonetti, 2019).

Here, to study septin biology in vivo, we characterise two septin null mutants in zebrafish, *sept6* ^−/−^ and *sept15* ^−/−^. We show that Sept6 and Sept15 null mutants are viable but are more susceptible to *S*. *flexneri* infection.

## 2 RESULTS

### 2.1 Generation of zebrafish Sept6 and Sept15 null mutants

As determined using RNAseq, zebrafish *sept6* and *sept15* are highly expressed at the whole animal level (Torraca et al., 2019; van Ngo et al., 2023). Previous knockdown work (using morpholino oligonucleotides) has indicated roles for Sept6 in early embryonic development (Zhai et al., 2014) and Sept15 in response to *S*. *flexneri* infection (Mazon‐Moya et al., 2017). To study the role of these genes in animal development and response to infection, we examined zebrafish harbouring a null mutant allele for *sept6* or *sept15*.

**FIGURE 1:**
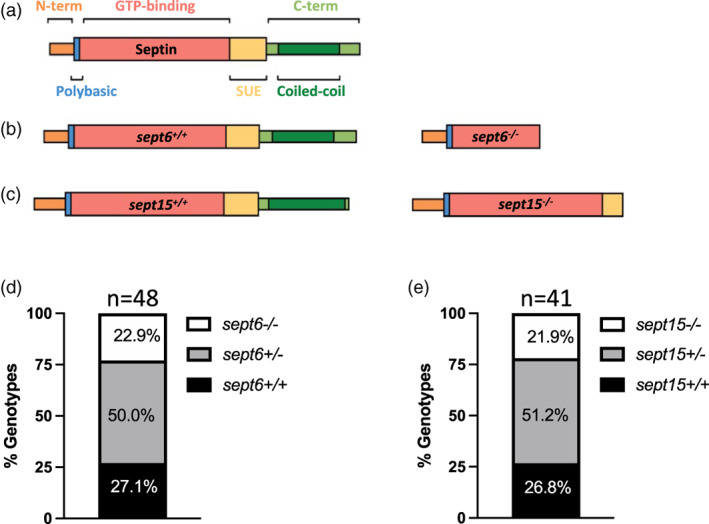
Generation of *sept6* and *sept15* mutants. (a) Schematic representation of the general septin protein structure and consequence of gene mutations at the level of the protein structure. (b,c) Specific representation of Sept6 (b) and Sept15 (c) protein structure and consequence of gene mutations at protein levels in Sept6 and Sept15 mutants. A *sept6* null allele was generated by CRISPR/Cas9 gene editing. A *sept15* null allele was identified from an ENU mutagenesis screen. (d,e) Genotyping of the adult offspring of *sept6* (d) and *sept15* (e) heterozygote carriers indicated no inheritance disequilibrium and homozygote mutants reached adulthood at the same rate as co‐housed wild‐type fish. The proportions of the different genotypes did not significantly differ from the attended Mendelian proportions by *chi*‐squared test. Sample size: a total of 48 (*sept6*) and 41 (*sept15*) adults (~6 months old) were genotyped

A Sept6 null mutant was generated by CRISPR/Cas9 mutagenesis. The selected mutant has a five nucleotide depletion in the *sept6* gene coding region, resulting in a frameshift (Figures 1a,b and S1). A Sept15 null mutant (allele *sa44249*) was generated by a random *N*‐ethyl‐*N*‐nitrosourea (ENU) mutagenesis screen carried out by the Zebrafish Mutation Project (https://zfin.org/ZDB-ALT-160601-9632; Kettleborough et al., 2013). This mutant has a single nucleotide change in the *sept15* gene coding region, resulting in a premature stop codon (Figures 1a,c and S1). In both Sept6 and Sept15 null mutants, the introduced mutations were designed to result in the production of truncated protein products and are predicted to lead to loss of function (Bowen, Hwang, Bai, Roy, & Spiliotis, 2011; Kim, Froese, Estey, & Trimble, 2011). The introduced mutations do not affect the Mendelian transmission of the mutant allele in heterozygote incrosses, and homozygote mutants reach adulthood (i.e., >6 months old) at the same rate as their wild‐type siblings (Figure 1d,e).

### 2.2 Characterisation of zebrafish Sept6 and Sept15 null mutants

**FIGURE 2:**
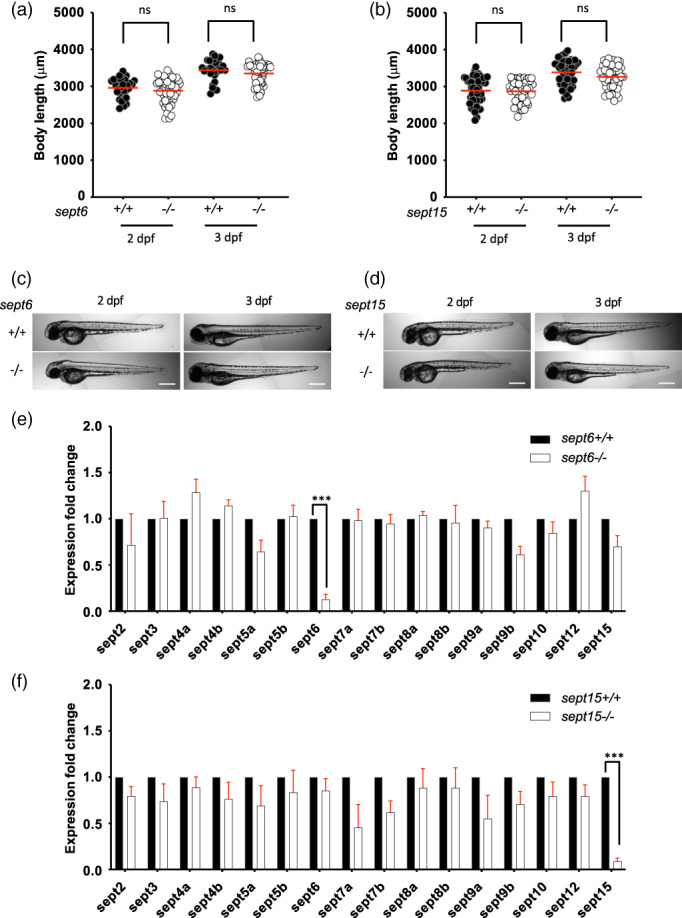
Characterisation of septin mutants. (a–d) *sept6* (a,c) and *sept15* (b,d) mutants do not have aberrant development. Comparison of the larvae length for *sept6* (a) and *sept15* (b) mutants to wild‐type larvae, at 2 and 3 dpf. The body lengths of mutants and wild‐types did not differ significantly. Representative images of *sept6* (c) and *sept15* (d) mutant and wild‐type larvae at 2 and 3 dpf. Scale bar: 500 μm. Results are cumulative of three independent experiments. Sample size: in total, 30 (wild‐type) and 48 (mutant) fish larvae were analysed for *sept6* and 48 (wild‐type) and 48 (mutant) fish larvae were analysed for *sept15*. Statistics: Kruskal–Wallis test; ns *p* > 0.05. (e,f) Both *sept6* (e) and *sept15* (f) homozygote mutations lead to nonsense‐mediated mRNA decay of the mutated transcript but do not significantly impact the expression level of other septins. Expression analysis was performed by qRT‐PCR, using primers able to amplify both mutant and wild‐type transcripts. Data are represented as expression fold changes against the expression level of the wild‐type group. Sample size: 15–20 larvae at 3 dpf were pooled together to extract mRNA from four independent replicates. Statistics: differences in gene expression levels were determined by multiple paired *t* tests, with Benjamini, Krieger, and Yekutieli to correct for false discovery (false discovery rate assumed 1%). ***p < 0.001

Using a high‐content screening approach, we tested larvae for Sept6‐ or Sept15‐dependent changes in development and morphology (Figure 2a–d). In wild‐type larvae, *sept6* mutants and *sept15* mutants, we observed an increase in whole body length from 2 to 3 days post fertilisation (dpf). For all larvae tested, the whole body length was similar at both 2 and 3 dpf.

The introduction of a premature stop codon and frameshift often results in nonsense‐mediated mRNA decay of the truncated gene (Behm‐Ansmant et al., 2007). Reports have indicated that the introduction of such mutations may result in genetic compensation, in which genes with high sequence homology (to that of the targeted gene) can be upregulated (El‐Brolosy et al., 2019). To assess if nonsense‐mediated mRNA decay occurred in our null mutants, and whether mutation of septin genes led to genetic compensation (and hence upregulation) of other septin genes, we performed qRT‐PCR for all zebrafish septin genes in *sept6* mutants, *sept15* mutants and their wild‐type siblings. We designed primers for the detection of *sept6* and *sept15* expression that did not overlap with the mutation site; in this way, primers can equally detect the expression level of mutant and wild‐type transcripts for their intended target (Dataset S1). We detected a significant reduction of *sept6* mRNA (CT values >34.99 in mutants vs. <33.65 in wild‐type) and *sept15* mRNA (CT values >27.71 in mutants vs. <25.96 in wild‐type) in the corresponding mutants (Figure 2e,f). However, in Sept6 and Sept15 mutants, no other septin was significantly upregulated or downregulated (Figure 2e,f), showing that other zebrafish septins do not compensate for the lack of Sept6 or Sept15. Together, our data indicate that the introduced mutations lead to low‐level mRNA expression of the targeted septin gene. We cannot rule out that low‐level truncated proteins may be still incorporated into septin complexes. However, it is unlikely that their incorporation into native septin complexes can occur without functional effects, considering that C‐terminal truncations of SEPT2 and SEPT6 have been previously reported to have functional effects on septin assembly and cytokinesis (Bowen et al., 2011; Kim et al., 2011).

### 2.3 Zebrafish Sept6 and Sept15 null mutants are more susceptible to *Shigella* infection

The *S*. *flexneri*‐zebrafish infection model was originally developed to study septin‐mediated immunity in vivo, highlighting a key role for the bacterial Type III Secretion System (T3SS) in virulence (Duggan & Mostowy, 2018; Mostowy et al., 2013). Previous work has shown that knockdown of Sept7b, Sept15 (using morpholino oligonucleotide; Mazon‐Moya et al., 2017) or Sept2 (using F0 CRISPR) (van Ngo et al., 2023) results in significantly increased susceptibility to *S*. *flexneri* infection.

**FIGURE 3:**
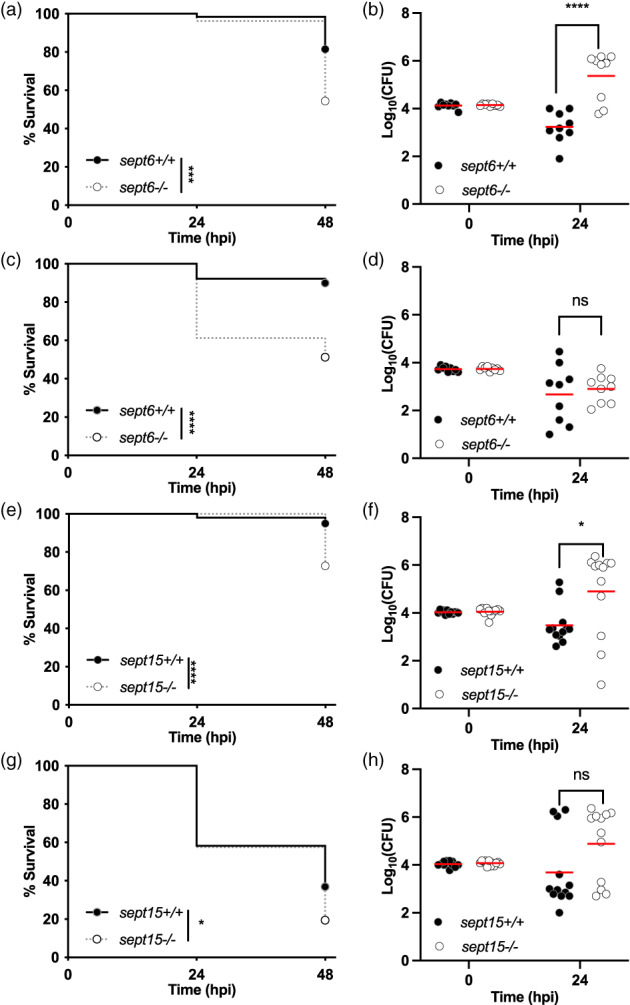
Zebrafish null mutants of Sept6 and Sept15 are more susceptible to *Shigella* infection. (a–d) Survival curves (a,c) and Log_10_‐transformed CFU counts (b,d) of 3 dpf *sept6* ^*+/+*^ and *sept6* ^*−/−*^ larvae (wild‐type in black; mutant in white) injected in the hindbrain ventricle (HBV) with *S*. *flexneri* GFP+ at a dose of 10,000 CFUs (a,b) or a dose of 5,000 CFUs (c,d). Next, larvae were incubated at either 28.5°C (a,b) or 32.5°C (c,d), for up to 48 h. Experiments are cumulative of three biological replicates. Sample size: For survival analysis, a total of 65 (wild‐type) and 79 (mutant) and 90 (wild‐type) and 80 (mutant) larvae was analysed at 28.5°C and 32.5°C, respectively. For CFU analysis, a total of 9 larvae was analysed per experimental group. Only living larvae were used for CFU enumeration. Statistics: Log‐rank (Mantel‐Cox) test (a,c); unpaired *t* test on Log_10_‐transformed values (b,d). ****p < 0.0001; ns *p* > 0.05, respectively. (e,h) Survival curves (e,g) and Log_10_‐transformed CFU counts (f,h) of 3 dpf *sept15* ^*+/+*^ and *sept15* ^*−/−*^ larvae (wild‐type in black; mutant in white) injected in the HBV with *S*. *flexneri* GFP+ at a dose of 10,000 CFUs. Next, larvae were incubated at either 28.5°C (e,f) or 32.5°C (g,h) for up to 48 h. Experiments are cumulative of four biological replicates. Sample size: For survival analysis, a total of 99 (wild‐type) and 104 (mutant) and 103 (wild‐type) and 113 (mutant) larvae was analysed at 28.5°C and 32.5°C, respectively. For CFU analysis, a total of 12 larvae was analysed per experimental group. Only living larvae were used for CFU enumeration. Statistics: Log‐rank (Mantel–Cox) test (e, g); unpaired *t* test on Log_10_‐transformed values (f,h). *p < 0.05; ****p < 0.0001; ns *p* > 0.05, respectively

To test the role of Sept6 or Sept15 in host defence, we used our *S*. *flexneri*‐zebrafish infection model and injected *S*. *flexneri* in the hindbrain ventricle of 3 dpf zebrafish larvae. The larvae were then incubated for 48 hr at either 28.5°C or 32.5°C. Previous work from our lab (using different *Shigella* and Enteroinvasive *E*. *coli* strains) has revealed temperature‐dependent and independent mechanisms of virulence, that can be distinguished by performing experiments at different temperatures (Miles et al., 2022; Torraca et al., 2019). For example, the T3SS is expressed in a temperature‐dependent manner and the use of two temperatures allows us to study the role of septins in the context of different levels of T3SS activation. Strikingly, both Sept6 and Sept15 null mutants are significantly more susceptible to *S*. *flexneri* infection than their wild‐type siblings at 28.5°C and 32.5°C (Figure 3a–h). Our data suggest that the wild‐type background (AB) of Sept6 null mutants is more susceptible to *Shigella* infection, as compared to the wild‐type background (TL) of Sept15 null mutants (Figure S2). We observed an increase in bacterial burden for septin null mutants at 24 hpi at 28.5°C (Figure 3b,f). At 32.5°C, the bacterial burden is not significantly different for wild‐type and null mutants at 24 hpi (Figure 3d, h), indicating that inflammation may underlie these survival differences (Mazon‐Moya et al., 2017). Overall, these results highlight an important role for Sept6 and Sept15 in host defence against *S*. *flexneri* infection.

## 3 CONCLUDING REMARKS

Here, we show that zebrafish *sept6* and *sept15* null mutants are viable and that other septins are not transcriptionally upregulated or downregulated in either of these two null mutants (suggesting that other septin genes are not compensating for the loss of *sept6* and *sept15*). The viability of zebrafish *sept6* ^*−/−*^ is consistent with data from the mouse model, where *Sept6* ^−/−^ mice are also viable (Ono et al., 2005). The viability of zebrafish *sept15* ^*−/−*^ (a homologue of mammalian SEPT7) is in contrast with data from the mouse model, where *Sept7* ^−/−^ mice are embryonic lethal (Ageta‐Ishihara et al., 2013; Hall, Russell, & Pringle, 2008; Menon et al., 2014). However, there is only one SEPT7 group member in the mouse genome, while zebrafish have three SEPT7 group members (Sept7a, Sept7b, and Sept15). Although we do not observe compensation for Sept15 deficiency by upregulation of other septins, increased copy number within the SEPT7 group may enable zebrafish to be viable in the absence of Sept15.

Our results also show that Sept6 and Sept15 are required for host defence against *S*. *flexneri* infection. Although septins have previously been implicated in cell‐autonomous immunity and cell death (Brokatzky & Mostowy, 2022; Mostowy & Cossart, 2012; Mostowy & Shenoy, 2015), the precise role of Sept6 and Sept15 in host defence in vivo is not known. These results highlight *S*. *flexneri* infection of zebrafish as a powerful system to study the role of individual septins in vivo. In the future, we expect that in vitro work using purified septin proteins (Lobato‐Márquez et al., 2021; López‐Jiménez & Mostowy, 2021) will be useful to investigate zebrafish septin hetero‐oligomer assembly and understand which zebrafish septins are essential for complex formation in vivo.

In conclusion, we show that zebrafish studies on septin biology can be used to complement those being performed in mice. Mice and zebrafish are vertebrates and the septin family in these models share close sequence homology (Shuman & Momany, 2022). Humans and mice have 13 septins and zebrafish 19 septins, representing the same four septin groups (SEPT2, SEPT3, SEPT6, and SEPT7), although, in the case of zebrafish, a 1‐to‐many homology is present due to gene duplication. In contrast, *Drosophila* has five septins and *Caenorhabditis elegans* has two septins, and these do not fully represent the four septin groups present in vertebrates. For example, *Drosophila* lacks a clear SEPT3 homologue and classification of *C*. *elegans* septins (UNC‐59 and UNC‐61) into septin groups is unclear. As a result, the higher‐order assembly of septin complexes is predicted to be different in vertebrates (hexamers, octamers), *Drosophila* (hexamers) and *C*. *elegans* (tetramers). We propose that in‐depth knowledge of septin biology obtained from complementary animal models can help develop innovative host‐directed therapies to combat bacterial infection in humans.

## 4 MATERIALS AND METHODS

### 4.1 Ethics statements

Animal experiments were performed according to the Animals (Scientific Procedures) Act 1986 and approved by the Home Office (Project licence: PPL P4E664E3C). All experiments were conducted up to 5 dpf.

### 4.2 Zebrafish husbandry

The *sept6* CRISPR mutant strain was made in the wild‐type AB background, while the *sept15* N‐ethyl‐N‐nitrosourea (ENU) mutant strain was originally produced in the wild‐type Tüpfel longfin (TL) background, within the framework of the Zebrafish Mutation Project (https://zfin.org/ZDB-ALT-160601-9632) (Kettleborough et al., 2013). Briefly, adult males were treated with the ENU mutagen, mutagenised sperm was collected and subsequently used for the in vitro fertilisation of a wild‐type egg pool, to generate an offspring of heterozygote carriers of random point mutations. In both cases, founders carrying the mutant alleles were identified by genotyping and outcrossed to wild‐type AB fish. Heterozygotes were incrossed to obtain all genotypes. Homozygote mutants and their wild‐type siblings were then maintained by inbreeding.

Unless specified otherwise, eggs, embryos and larvae were reared at 28.5°C in Petri dishes containing embryo medium, consisting of 0.5× E2 water supplemented with 0.3 μg/mL methylene blue (Sigma‐Aldrich, St. Louis, MO). For injections, anaesthesia was obtained with buffered 200 μg/mL tricaine (Sigma‐Aldrich) in embryo medium. Protocols comply with standard procedures as reported at zfin.org. Genotyping was performed on individual larvae using KASP genotyping assays (LGC Biosearch Technologies). Primers used for the assays are reported in Table 1.

**TABLE 1:** Oligonucleotide sequences used in this study.

Application	Oligo name	Sequence
qRT‐PCR	sept2qFw	TGGTTACATTCTGCCTCTGTCG
qRT‐PCR	sept2qRv	GCTCAAACAAGTCAGCATCGTG
qRT‐PCR	sept3qFw	GCTACCAAAACAGGGATCGACAT
qRT‐PCR	sept3qRv	ATGAGAGGGCTTAGCTGGGA
qRT‐PCR	sept4aqFw	CTCTCGCCCCAAAAGTCCAT
qRT‐PCR	sept4aqRv	TAAACCGGACTCTCCTGCCA
qRT‐PCR	sept4bqFw	AGGAGCAGAGACGCGATGATG
qRT‐PCR	sept4bqRv	GCGTGGACTTCCCTAAACCTG
qRT‐PCR	sept5aqFw	CACCATTTGGACACGGTTTGC
qRT‐PCR	sept5aqRv	CCCGAAACGCTCAATCTCGT
qRT‐PCR	sept5bqFw	GGTGAAATCAGAGGATGGAGAGG
qRT‐PCR	sept5bqRv	AGTGTGGATTTCCCTAAGCCAG
qRT‐PCR	sept6qFw	TGAGACACCTTATGCTGCTTCTTT
qRT‐PCR	sept6qRv	CAAGGCTGACGGAGTGACAA
qRT‐PCR	sept7aqFw	AGGTGGAGCAATCCAAGGTG
qRT‐PCR	sept7aqRv	CGTGACTCCGCGTTAAGGTA
qRT‐PCR	sept7bqFw	CAGGACATGGGCTGAAACCTT
qRT‐PCR	sept7bqRv	GGGGTCAGCGTATCTGCTTT
qRT‐PCR	sept8aqFw	CATCCTCTGTGTGGGTGAGAC
qRT‐PCR	sept8aqRv	CCGTTTTGGTAATGGCTGGC
qRT‐PCR	sept8bqFw	ACATATCCAACTGAGGAGATGCG
qRT‐PCR	sept8bqRv	TTGACTGACTGATTTGCTGACCA
qRT‐PCR	sept9aFw	AACCATCGAGATCAAGTCCGTC
qRT‐PCR	sept9aRv	TGGGCTGCCAACAATTCTCA
qRT‐PCR	sept9bqFw	ACCACACATTGCGAGTTTGC
qRT‐PCR	sept9bqRv	CGAACGCGGTACATCTCGTA
qRT‐PCR	sept10qFw	GAGTGTAAACCTGTCAGTCTCCAG
qRT‐PCR	sept10qRv	CGTCTCTGCAGTTCACCCAG
qRT‐PCR	sept12qFw	TGGAAGTTTTGCCTGGACTGAA
qRT‐PCR	sept12qRv	TCTTCAGAGCCTCAAAATCAGACAA
qRT‐PCR	sept15qFw	AAACTGATCCGCAAGATAAAGGAGA
qRT‐PCR	sept15qRv	CAGTGCTCACCGTTCTCCAC
CRISPR	sept6_81mer	GCGTAATACGACTCACTATAGGGACTTAAGCGAATGTCCAGGTTTTAGAGCTAGAAATAGCAAGTTAAAATAAGGCTAGTC
CRISPR	univ_81mer	GATCCGCACCGACTCGGTGCCACTTTTTCAAGTTGATAACGGACTAGCCTTATTTTAACTTGCTATTTCTAGCTCTAAAAC
CRISPR	T7_ampl	GCGTAATACGACTCACTATAG
CRISPR	univ_ampl	GATCCGCACCGACTCGGT
Genotyping	sept6KASPx	GAAGGTGACCAAGTTCATGCTCTGTATTTCATTGCTCCCACT
Genotyping	sept6KASPy	GAAGGTCGGAGTCAACGGATTGTATTTCATTGCTCCCACA
Genotyping	sept6KASPc	CATAGTCACCAGGTCAAGGGACTTAA
Genotyping	sept15KASPx	GAAGGTGACCAAGTTCATGCTAGGACGTGACCAATAATGTTCACTAC
Genotyping	sept15KASPy	GAAGGTCGGAGTCAACGGATTAAGGACGTGACCAATAATGTTCACTAA
Genotyping	sept15KASPc	GGCTGCTAGTTTCTTACTGCGGT

### 4.3 Zebrafish CRISPR‐Cas9 editing

The *sept6* ^*−/−*^ zebrafish line was generated using CRISPR‐Cas9 technology following a method previously described in Sommer, Torraca, Kamel, Lombardi, and Meijer (2020). Briefly, a short guide RNA (sgRNA) targeting the proximal region of the *sept6* gene (ENSDARG00000010721) was designed using the chop–chop web server (https://chopchop.cbu.uib.no/). The sequence of the CRISPR target +PAM used was GGACTTAAGCGAATGTCCAGTGG. To obtain the sgRNA, the partially complementary 81‐mer DNA oligonucleotides sept6_81mer and univ_81mer were annealed together and used as a template for a PCR reaction, using primers T7_ampl and univ_ampl, resulting in a 122 bp product that could be used as a template for sgRNA synthesis, using MEGA short script® T7 kit (AM1354; ThermoFisher). The sequences of oligonucleotides used are reported in Table 1.

The Cas9 was delivered as an mRNA. For this, a zebrafish‐optimised NLS‐Cas9‐NLS was transcribed using the mMACHINE® SP6 Transcription Kit (AM1340; Thermo Fisher) from a Cas9 plasmid (47929, Addgene).

Embryos were injected with a 1 nL mixture containing 150 pg sgRNA/150 pg NLS‐Cas9‐NLS mRNA at 0 hpf and CRISPR injections were confirmed by PCR and Sanger sequencing. One founder (F0) was outcrossed with AB fish to propagate the mutated allele. The chosen mutation consists of a 5 bp deletion targeting the GTP‐binding domain of Sept6. A stable line was generated by incrossing heterozygous F1 carriers.

The *sept15* ^*−/−*^ zebrafish line is a homozygote carrier of the *sa44249* allele, which was generated by the Zebrafish Mutation Project (Kettleborough et al., 2013). Heterozygote carriers were first obtained from in vitro fertilisation of TL eggs with the sperm of *sa44249* allele carriers. These were outcrossed to AB, then the offspring was incrossed to obtain homozygote mutants.

### 4.4 Quantitative RT‐PCRs

qRT‐PCRs were performed using a 7500 fast real‐time PCR System machine and 7500 software v2.3 (Applied Biosystems, Foster City, CA) and a SYBR green master mix (Applied Biosystems). Briefly, RNA was isolated from pools of whole larvae at 3 dpf using RNeasy mini kit (Qiagen, Hilden, Germany). cDNA was obtained using a QuantiTect reverse transcription kit (Qiagen). Samples were run in biological quadruplicates and quantification was obtained using the 2^*−*ΔΔCT^ method and *eef1a1a* as a housekeeping gene. Table 1 reports all primers used in this study. Primers for *sept6* and *sept15* did not overlap with the mutation sites for their corresponding mutant and can detect the expression level of both the mutant and wild‐type transcript variants of their target (Dataset S1).

### 4.5 Zebrafish larvae whole length body measurements

Dechorionated zebrafish larvae 2 dpf were first anaesthetised (as described above) by immersing them in methylene blue‐free embryo medium containing tricaine (methylene blue was omitted to obtain better imaging) and then single larvae were distributed into each well of a 96‐well plate (Perkin‐Elmer). Wells containing larvae were topped‐up with tricaine‐containing embryo medium without methylene blue. Larvae were positioned on their side in the middle of each well (ready for imaging), and the 96‐well plate was inserted into the Zeiss CellDiscoverer 7 (CD7) high content/high throughput automated imaging system. Using the APEER (Zeiss) platform (www.apeer.com) we designed and trained an AI model to identify zebrafish larvae at different developmental stages in brightfield images acquired with a widefield microscope. This model was then used within the ZEN Blue software (v3.5) to automatically image the 96‐well plate and outline the larvae in the acquired image. When completed, the 96‐well plate was removed from the microscope, embryo medium was replaced with medium not containing tricaine, and larvae were left in an incubator overnight at 28.5°C. The next day, the same larvae (now 3 dpf) were again anaesthetised (as on the previous day), scanned and images processed as described above. The measurements of the larvae's whole body length (width of the larvae outline) were automatically generated and displayed in an excel file. GraphPad software was used to generate the final results.

### 4.6 Bacterial infections

GFP fluorescent or nonfluorescent *S*. *flexneri* M90T was used for all zebrafish infections. Bacterial preparation and infection were performed as previously described (Torraca et al., 2019). Briefly, bacteria were grown to log phase, spun down, washed in PBS, and resuspended to a density equivalent to an OD600 = 10 (for 5,000 CFU) or 20 (for 10,000 CFU) in an injection buffer containing 2% polyvinylpyrrolidone (Sigma‐Aldrich) and 0.5% phenol red (Sigma‐Aldrich) in PBS. 1 nL (corresponding to 5,000 or 10,000 CFU) of bacterial suspension was microinjected in the hindbrain ventricle (HBV) of zebrafish larvae 3 dpf. Bacterial enumeration was performed a posteriori by mechanical disruption of infected larvae in 0.4% Triton X‐100 (Sigma‐Aldrich) and plating of serial dilutions onto Congo red‐TSA plates.

### 4.7 Statistics and data presentation

Statistical analysis and graphs were created using GraphPad software. Mendelian distributions of genotypes were tested by *chi*‐squared test (Figure 1d,e). Body lengths were tested by Kruskal–Wallis test, separately at 2 and 3 dpf (Figure 2a,b). Statistical differences in gene expression levels were determined by multiple paired *t* tests, with Benjamini, Krieger and Yekutieli to correct for false discovery (false discovery rate assumed 1%; Figure 2e,f).

Statistical differences in survival were tested by Log‐rank (Mantel–Cox) test (Figures 3a,c,e,g and S2A).

Statistical differences in bacterial load were tested by unpaired *t* test on Log_10_(CFU) values (Figures 3b,d,f,h and S2B).

## AUTHOR CONTRIBUTIONS

Serge Mostowy conceived and supervised this study. Vincenzo Torraca, Magdalena K. Bielecka, Margarida C. Gomes, Dominik Brokatzky, Elisabeth M. Busch‐Nentwich and Serge Mostowy designed the experiments. Vincenzo Torraca, Magdalena K. Bielecka, Margarida C. Gomes and Dominik Brokatzky performed experiments. All authors performed data analysis, and took part in the interpretation of results and preparation of materials for the manuscript. Vincenzo Torraca and Serge Mostowy wrote the manuscript with comments from all authors.

## CONFLICT OF INTEREST STATEMENT

The authors declare no conflicts of interest.

## Supporting information


**Dataset S1.** Annotated cDNA sequences for zebrafish *sept6* and *sept15*. Relevant sequences and sites are annotated in different colours. Sequences highlighted in grey: 3′ and 5′‐untranslated regions (UTR); sequences highlighted in green and yellow: qRT‐PCR forward and reverse priming sites, respectively; Sequences highlighted in red: mutation sites; bold, underlined sequences in black: start and wild‐type stop codons; bold, underlined sequences in blue: premature stop codon position created by the mutation.

**FIGURE S1.** Confirmation of *sept6* mutation by sequencing and details of *sept15* mutation. (a) Confirmation of *sept6* mutation on a heterozygote carrier using MiSeq analysis. The predicted cleavage position represents the position where the Cas9 is expected to introduce a double‐strand DNA break, in agreement with the short guide RNA sequence we designed. As expected the introduced 5‐nucleotide deletion encompasses the intended target region. (b) Expected effect of *sept6* mutation at the protein sequence level. The sequence highlighted in a black box corresponds to the peptidic sequence created by the frameshift and is nonhomologous to the wild‐type Sept6 protein sequence. (c) Specifications of *sept15* sa44249 mutant allele, as retrieved from zfin.org (https://zfin.org/ZDB-ALT-160601-9632). (d) Expected effect of *sept15* mutation at the protein sequence level.

**FIGURE S2.** An input of 10,000 CFU is highly lethal for *sept6* mutants at a higher temperature. Survival curves (a) and Log_10_‐transformed CFU counts (b) of 3dpf *sept6+/+* and *sept6−/−* larvae (wild‐type in black; mutant in white) injected in the HBV with *S*. *flexneri* GFP+ at a dose of 10,000 CFUs. Next, larvae were incubated at 32.5°C for up to 48 h. Experiments are cumulative of two biological replicates. Sample size: for survival analysis, a total of 56 (wild‐type) and 55 (mutant) larvae was analysed at 32.5°C. For CFU analysis, a total of six larvae was analysed per each experimental group. Only living larvae were used for CFU enumeration. Statistics: Log‐rank (Mantel–Cox) test (A); ns *p* > .05; Plating at 24 hpi was not done as there were no live zebrafish at the 24 h timepoint.

## Data Availability

The data that support the findings of this study are available from the corresponding author upon request.
