# Supplementary material for: Zebrafish null mutants of Sept6 and Sept15 are viable but more susceptible to Shigella infection
Source: Cytoskeleton (Hoboken). 2023 Mar 14;80(7-8):266–74. doi: 10.1002/cm.21750 (PMC10952258; doi:10.1002/cm.21750)
Supplement: Supplementary file 1 — Dataset S1. Annotated cDNA sequences for zebrafish sept6 and sept15. Relevant sequences and sites are annotated in different colours. Sequences highlighted in grey: 3′ and 5′‐untranslated regions (UTR); sequences highlighted in green and yellow: qRT‐PCR forward and reverse priming sites, respectively; Sequences highlighted in red: mutation sites; bold, underlined sequences in black: start and wild‐type stop codons; bold, underlined sequences in blue: premature stop codon position created by the mutation. [file CM-80-266-s002.docx]

**Supplementary Dataset S1. Annotated cDNA sequences for zebrafish *sept6* and *sept15***

***Sept6* cDNA sequence**

GGTACAGTCCATCGTATGTGAGTAGTCGCTGTAGCTTCAGTTTGCGTGCTGCTGCAGTTTCCTGTGAAGGTTTG**ATG**CAGGAGAGAACCATGGCGGCCACTGAGATAGCACGACAAGCGGGAGAGGGTGCACGTGCTGTCCCACTCTCTGGTCATGTTGGCTTTGACAGCATGCCGGACCAGCTGGTCAACAAGTCCGTCAACCATGGTTTCTGCTTCAATATCCTCTGTGTGGGGGAGACGGGTTTGGGAAAGTCAACCCTCATGGACACCCTGTTTAACACCAAATTTGAGGGCGAACCTACACAGCACAATCAGCCTGGAGTGCAGCTCAAATCCAACACTTATGAGCTGCAGGAGAGTAATGTCCGACTCAAGCTCACTGTGGTCAACACTGTAGGATTTGGAGACCAGATCAACAAAGAGGACAGTTACAAGTCTATTGTGGAGTTCATCGATGCTCAGTTTGAAGCGTACCTTCAGGAGGAACTGAAGATTAAACGCACACTACACAGTTATCATGATACACGGATCCACGCTTGCCTGTATTTCATTGCTCCCACTGGACATTCGCT**TAA**GTCCCTTGACCTGGTGACTATGAAGAAGTTGGACAGTAAGGTAAATATCATCCCCATCATTGCCAAATCAGATGCCATTTCAAAGAGTGAACTTGCCAAGTTCAAAATCAAGATCACGAGTGAGTTGGTGAGCAATGGCGTCCAGATCTACCAGTTTCCCACTGATGATGAGACCGTGGCGGAGATCAACTCAACTATGAATGGTCATTTGCCTTTTGCAGTGGTGGGAAGCACTGAGGAAGTGAAGATTGGGAACAAGATGGTGCGAGCACGCCAGTACCCATGGGGAACCGTCCAGGTGGAGAATGAGAATCACTGTGATTTCGTGAAGCTGAGAGAGATGCTGATCAGGGTCAACATGGAGGACCTGCGGGAGCAGACCCACACTCGCCATTATGAGCTTTATCGCCGTTGCAAACTGGAAGAGATGGGATTCAAAGACACTGACCCCGATAGCAAACCTTTCAGCCTTCAGGAAACATATGAGGCCAAGAGGAATGAGTTCATGGGTGAGCTTCAGAAGAAAGAGGAGGAGATGAGGCAGATGTTTGTCCAGAGAGTCAAAGAGAAGGAGGCAGAGCTGAAAGAGGCAGAAAAGGAGCTGCATGAGAAGTTTGATCGCCTCAAGAAACTTCACCAGGACGAGAAGAAGAAACTGGAGGACAAGAAGAAGTCTCTTGATGATGAGCTGAACGGCTTCAAGCAGAAGAAAACTGCTGCCGAGCTGCTGCAGTCACAGAACCAGCAGCCAGGGGGCTCCGCCACACTCAAGAAGGACAAAGAGAGGAAAAAT**TAA**CTCCTCACCTGCTCCCAGTCCTGCATGAGACACCTTATGCTGCTTCTTTTAACTCTGTCTTGGCCACCTGTTGGAAGAGGGGATCGCAGCTTCCACAAGAACTAAGCATCCGCTCTTCTTCCTGTCTTTGTCACTCCGTCAGCCTTGTTTGAATGTTTTATTTAATTTTTTTATTTCATGCTGTGTGCCCTGTTAAACATGGCCTTATGTGGGCTTAACATGGTATTTTTCAACAAATATTAGCTCTGTTTTTTAATTGGGGTTTGCTAAGCAGTCCCTCTTTAGTATTTTTACAGGCATCATTTGCTGGTGACAACAGAGCCAAAGCTCTGGTAGAAAGGCTGTGAAGTAAAGAAGTGTTTAAGTTTAGCTTTTTGCATCTGTGATATTGCCTTGCAGTCTCAATCAACCAATCATTTAGTTGCTTTTACGAGTTCATGTGTTTGTGAATGAACCTGCATGTGAAGATTAACACAGTTTTAACTGTGAAGAATGAATGCATACTAACTTATTTGCAAACTCTACATTTTAGGATGTGAACTAAGTTCAGCAAGATTATCTTTAATAAGACTTTTTGACACACTGGCTGTGAATATTGAGCAAATCCAAAGACAATGGGCCTGAACGTTATTGCTTGCTAATGCAGCAAAATCTTAAGTCAGAGATATTTAAGGAGTTTATTTGTTTTATCTGAGAGCATGAGTTAAATAGAAGCAAAATCTGAGAGGTTTGTATTGTATGTTTTGTAATGCCCTATACATTTTACAGCACACATTTCAAGTCTTAAATAGTCACAGCAATATAGTTACACTTATTTTAATTATATTATCTTCGAGAGTAATAGAGCATCTATTGAAATACCATGTAGGTCACATTCAGTGCCATTTTTATATTCTTTATCTTCTGTTCTTCTGCATGGCCTGAACATTTATATTTAATTTCTTATTTCGTTTTGTTTATATAGAGAGTTAATCTTTACTTTGACATATTTACTCTTCTTTGGATCATTTATTCTAGGTACAGTGTGCATTCAGTTTCTAAATGGAATTTCCCATTTTGCCAAATATTGCATTTAGATATACGTCAAGGTTTTTTTTTTTTTTTGTCATTTAATGCAAGTCAGTTACGAGGTACAGATGTTCATGCATATGTGCAGTACATATACTTTTTTTCAGGACCTATTTTGCGTATCAGTGTTGAGACATGTTTCAATGATTATTGACAAGTGACTGTTTTCTGTGTAAAACATTTTGAGTGTCAAAACTGGTCTACATTTTTCTAGTCACCTTCTACATTTCCCCATTTATGTCAATTACCCTCATCCTTGTAATACCAGAAATTTGTGAAATATTTATCTGTAGTAAGATTCCATGGTGTACATTTTCAATAAAATGACAATAAATCAA

**Labels**

3’ UTR and 5’UTR

**START and wildtype STOP codon**

**Premature STOP codon position created by the mutation**

Wildtype nucleotides, depleted in the mutant

qRT-PCR Forward priming site

qRT-PCR Reverse priming site

***Sept15* cDNA sequence**

ATAGGAGTAGATCTCTCCCGGAGGAGGACAATCGCCTTTCATGCTCGCAGCGTTGTAACATTTTTCCCCCGGCCGATGCTCGCATGCCTGTTTTCGTCCCCTGCACGTTTACCTTCCCCAAAAAGCCATTAATTTAGTCCAAGCCCGAGTGCGCCGTAAGGGTTCGTCTTCTGCTCTCGACCGCTTTTTATTAGAGTCGACTCATCTTTTTATGCCTGATCTATTGGGATTCAGAGTGGGTGTTGTCACAGGACA**ATG**ATCGAGAGACCCGACTCAGCTGTGTCCAGCGTTGCACAGAGGAATCTGGAGGGTTATGTTGGATTCGCCAACCTCCCCAACCAGGTGTACAGGAAATCTGTGAAGAGGGGCTTCGAGTTCACGCTCATGGTTGTCGGTGAGTCTGGACTGGGCAAATCAACGCTCATCAATTCCCTCTTCCTGACAGACCTGTATTCCAAAGACTACCCTGGACCATCTCAGAGGATCAAGAAGACTGTTCAGGTTGAACAGTCCAAAGTGCTGATAAAGGAGGGGGGCGTCCAGCTCACACTCACCATCGTCGACACACCAGGATTTGGAGATGCGGTGGACAACAGCAACTGCTGGCAGCCTGTCATCAACTACATCGACAGTAAGTTTGAAGACTTCCTGAATGCTGAATCCCGTGTAAACAGGAGGCAGATGCCTGACAACAGGGTGCACTGCTGCTTGTACTTCATCGCCCCCTCTGGTCACGGACTGAAGCCTCTTGATATCGAGTTCATGAAGCGTCTGCATGATAAAGTCAATGTGATTCCTCTGATCGCCAAGGCAGATACACTGACGCCAGAAGAGTGTCAGCTCTTCAAGAAACAGATTATGAAGGAGATCCAGGAACACAAAATCAAGATCTACGAGTTTCCAGACACGGAGGACGACGAGGACAGCAAACTGATCCGCAAGATAAAGGAGAAGATGCCTCTGGCTGTGGTGGGCAGTAATGTGGTGATTGAAGTCAATGGCAGGAAGGTCAGAGGACGTCAGTACCCCTGGGGTGTGGCAGAAGTGGAGAACGGTGAGCACTGTGACTTCACAGTCCTAAGGAATATGCTCATCAGGACTCACATGCAGGACCTGAAGGACGTGACCAATAATGTTCAC**TA(C/A)**GAAAACTACCGCAGTAAGAAACTAGCAGCCGTCACCTGCAACGGGGTCGATGCCACCAAGAACAAAGGCCAGCTTACAAAGAGTCCACTGGCCCAGATGGAGGAGGAGAGGAGGGAGCATGTGATGAAGATGAAGAAGATGGAGACTGAGATGGAGCAGGTCTTTGAGATGAAGGTCAAAGAAAAGAAGCAAAAACTGAAGGACTCTGAGGCAGAGTTGGAACGGCGTCACGAACAGATGAAGAAGAATCTGGAAGCTCAGTATAAAGAGCTCGAGGAAAAGAGACGCCAGTTTGAGGATGAGAAAGCCAACTGGGAGGCGCAGCAGCGCATCCTGGAGCAGCAGAAGCTTGATGCATCAAAGACAATGGAAAAGAACAAGAAAAAAGGAAAAATCTTT**TAA**AGTCGTCATAAAGTTCCATTCCTCTTAAAACCACAAACTCCTTGCATCTGTTTTGATGCTCAGATGTTTTATGACCCCAGAAGAAACGCATGCACGAGTGCTACTGTACAGTTTAAATATAATAAAACCTTTACAGTCACAACCACACAACTTCATCGCCCTCTTCTGGTTTACCATAATATTACAGCACATTCCCCGTATGAGCATATTGACCAACACTCCATCAAAACATAGTATTTGTAATAATTTATGACTGTTTCTGGATGTTTGAAGGAGTCACGGTAGGTTTGGATGTGTGTGTGAGGGAGAATAAGAAAGTGAAAGCGAGCATGTACATGGTTAGGCACGTATGCATTGACGAGCAAAGCTACCGAGTGATGTATTTACGGACTGTGTGTGTTTCCTTGGGGTCATAAACATCCGTTTTAACAATATTGCACAGCTAAATTATAACAATAACAGTTTTCCATGTTTTTTAAACCCATTCTCTGGCTGTGTACGTAGATAATTGTGTCCCTACCATAGATGGTGTTCTATAAAAACGGCATGATTTTCAACATTAATAGTGGATTTTTTTTATTTGTATTAATACGGATGAGTATGAAATAATTACGATAATATTTTAGGATGTTGCTCGAACACTCTGCCTTAAATTTGCGGACTGTTTCGGCATTTGTCTGCTCATGTTTTTATTATTACTTTAAAAATTGTGAACCGATGTTCCTCAATCTGTTCCTAAACTGATCCGAGACCAGTTTGTTTCGACTTAATATACGTTTTAGGGCTGGTCCAGGATCAGGACTTCAGTATGTATATGAACAGATGTTAATTTTAGCCATACCCCAACTGGTTTTACTTTGCAGGTGTAAATGAGAAGCGCTCCTTTATTTTTTTTATGGATGGTTTAGATTGAAACTCGTGCACAAAACTCTCAATCTAAAGGGCAATAAACAGCCTTTACGCCTCATGTGATGGTATAATATGATATAATTTGCTGGTGTGTCTTTATGCAAGTTTGAACACTCAGTTCTTCCTGTCGTGGATGGTCAAACCACACAGAGGGATTTTAGATTCTGAATAAAAAGGATTTTTTTGTTAAACCACTCTACTTTTTTTAAATAGCAGACTTGAACATGACTCTAGACATGTTTGTTTTATTGTTATTATTATGCTTTTTTTGTATATGTATACGGTTTTTGAATTCCGAAGTTTCTTTTGTTTCCTTCACGTTATCTTCATCTGTTATTCTTTCTGTCTAACTGTACATTATTAAACATGAAATGCTGATAATG

**Labels**

3’ UTR and 5’UTR

**START and wildtype STOP codon**

**Premature STOP codon position created by the mutation**

Wildtype nucleotide, changed C>A in the mutant

qRT-PCR Forward priming site

qRT-PCR Reverse priming site
