# Supplementary material for: Zebrafish null mutants of Sept6 and Sept15 are viable but more susceptible to Shigella infection
Source: Cytoskeleton (Hoboken). 2023 Mar 14;80(7-8):266–74. doi: 10.1002/cm.21750 (PMC10952258; doi:10.1002/cm.21750)

FIGURE S1

A *sept6* CRISPR mutation

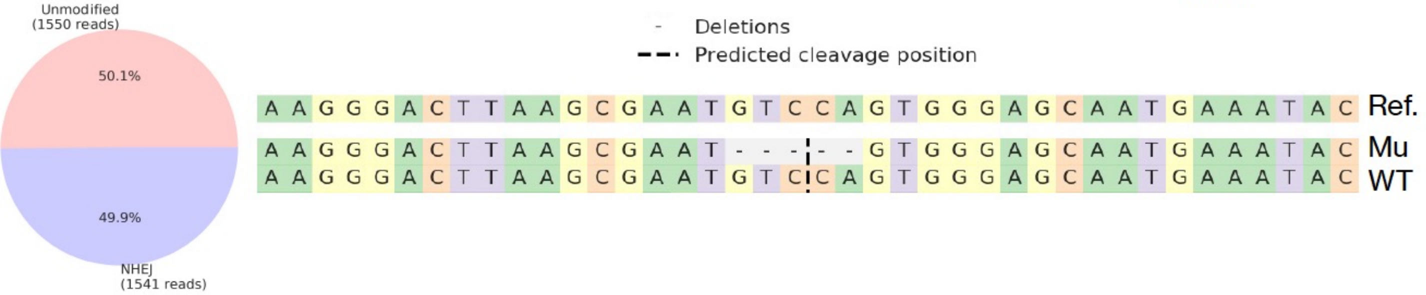

B

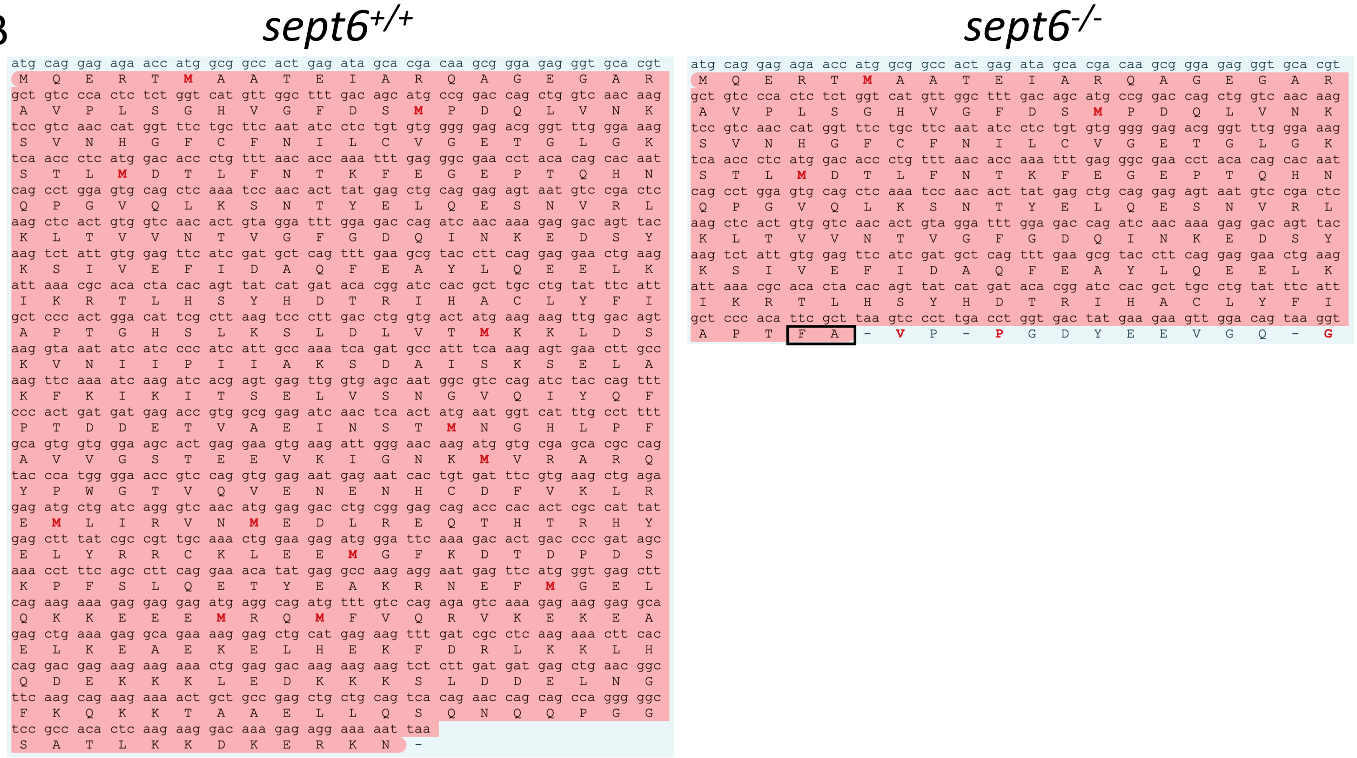

FIGURE S1 - CONTINUED

C *sept15* ENU allele *sa44249*

Variants

**Variant Type**	Point Mutation
**Variant Location**	Chr 25: 12859298 (GRCz11) (1) Details
**Nucleotide change**	G/T
**Variant Notes**	*None*

Effect on DNA/cDNA, transcript, protein

**DNA/cDNA Change**	C>A (1)
**Transcript Consequence**	Premature Stop (1)
**Protein Consequence**	*None*

D

sept15$^{+/+}$

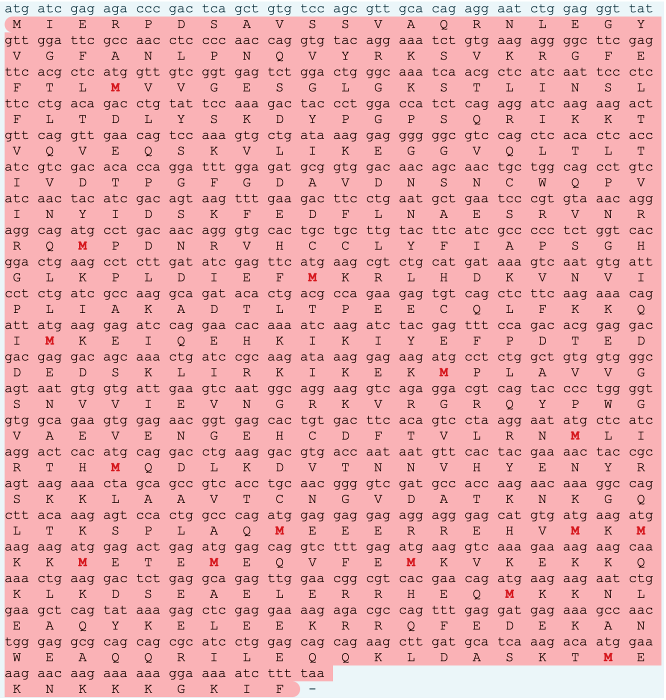

sept15$^{-/-}$

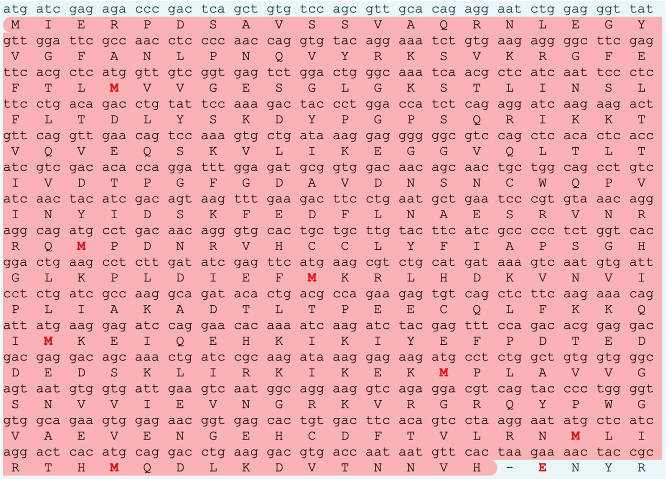

Supplement: Supplementary file 2 — FIGURE S1. Confirmation of sept6 mutation by sequencing and details of sept15 mutation. (a) Confirmation of sept6 mutation on a heterozygote carrier using MiSeq analysis. The predicted cleavage position represents the position where the Cas9 is expected to introduce a double‐strand DNA break, in agreement with the short guide RNA sequence we designed. As expected the introduced 5‐nucleotide deletion encompasses the intended target region. (b) Expected effect of sept6 mutation at the protein sequence level. The sequence highlighted in a black box corresponds to the peptidic sequence created by the frameshift and is nonhomologous to the wild‐type Sept6 protein sequence. (c) Specifications of sept15 sa44249 mutant allele, as retrieved from zfin.org (https://zfin.org/ZDB-ALT-160601-9632). (d) Expected effect of sept15 mutation at the protein sequence level. [file CM-80-266-s003.pdf]
