# Supplementary figures and images for: Zebrafish null mutants of Sept6 and Sept15 are viable but more susceptible to Shigella infection
Source: Cytoskeleton (Hoboken). 2023 Mar 14;80(7-8):266–74. doi: 10.1002/cm.21750 (PMC10952258; doi:10.1002/cm.21750)

FIGURE S2

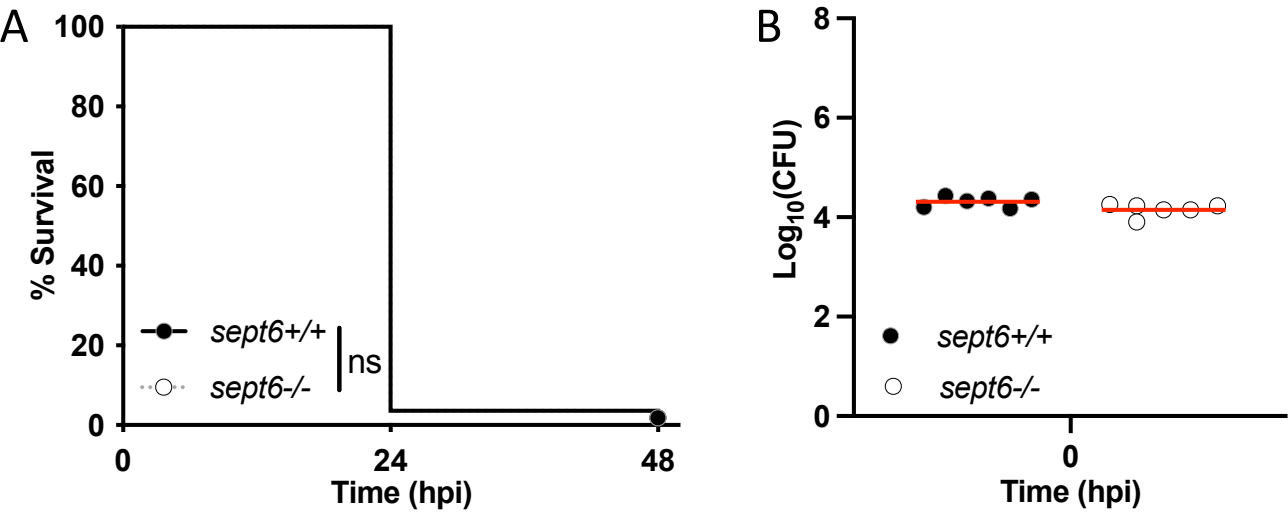

Supplement: Supplementary file 3 — FIGURE S2. An input of 10,000 CFU is highly lethal for sept6 mutants at a higher temperature. Survival curves (a) and Log10‐transformed CFU counts (b) of 3dpf sept6+/+ and sept6−/− larvae (wild‐type in black; mutant in white) injected in the HBV with S. flexneri GFP+ at a dose of 10,000 CFUs. Next, larvae were incubated at 32.5°C for up to 48 h. Experiments are cumulative of two biological replicates. Sample size: for survival analysis, a total of 56 (wild‐type) and 55 (mutant) larvae was analysed at 32.5°C. For CFU analysis, a total of six larvae was analysed per each experimental group. Only living larvae were used for CFU enumeration. Statistics: Log‐rank (Mantel–Cox) test (A); ns p > .05; Plating at 24 hpi was not done as there were no live zebrafish at the 24 h timepoint. [file CM-80-266-s001.pdf]
